# Propofol toxicity in the developing mouse heart mitochondria

**DOI:** 10.1038/s41390-022-01985-1

**Published:** 2022-02-16

**Authors:** Matthew B. Barajas, Sarah D. Brunner, Aili Wang, Keren K. Griffiths, Richard J. Levy

**Affiliations:** 1 Department of Anesthesiology, Columbia University Medical Center, New York, NY USA; 2 Department of Pediatrics, Division of Pediatric Critical Care Medicine, Columbia University Medical Center, New York, NY USA

## Abstract

**Background:**

Propofol infusion syndrome (PRIS) is a potentially lethal consequence of long-term propofol administration. Children are vulnerable and cardiac involvement is often prominent and associated with mortality. We aimed to determine the mechanism of propofol toxicity in newborn mice, hypothesizing that propofol would induce discrete defects within immature cardiac mitochondria.

**Methods:**

Newborn murine cardiac mitochondria were exposed to propofol or intralipid in vitro. Non-exposed mitochondria served as controls. Mitochondrial respiration and membrane potential (ΔΨ) were measured and respiratory chain complex kinetics were determined.

**Results:**

Propofol and intralipid exerted biological activity in isolated mitochondria. Although intralipid effects were a potential confounder, we found that propofol induced a dose-dependent increase in proton leak and caused a defect in substrate oxidation at coenzyme Q (CoQ). These impairments prevented propofol-exposed cardiomyocyte mitochondria from generating an adequate ΔΨ. The addition of the quinone analog, CoQ_0_, blocked propofol-induced leak and increased Complex II+III activity.

**Conclusions:**

Propofol uncoupled immature cardiomyocyte mitochondria by inducing excessive CoQ-sensitive leak and interfered with electron transport at CoQ. The findings provide new insight into the mechanisms of propofol toxicity in the developing heart and may help explain why children are vulnerable to developing PRIS.

**Impact:**

- Propofol uncouples immature cardiomyocyte mitochondria by inducing excessive coenzyme Q (CoQ)-sensitive proton leak.
- Propofol also interferes with electron transport at the level of CoQ.
- These defects provide new insight into propofol toxicity in the developing heart.

## Introduction

Propofol (2,6-diisopropylphenol) is the most common intravenous sedative–hypnotic used for general anesthesia.^1–3^ Because of its desirable pharmacokinetic properties, propofol is also widely utilized for procedural sedation in ambulatory settings and for non-procedural sedation of the critically ill.^2^ Propofol infusion syndrome (PRIS) is a potentially fatal adverse consequence that manifests with metabolic acidosis, arrhythmias, profound cardiac failure, rhabdomyolysis, hyperkalemia, hepatomegaly, hyperlipidemia, renal failure, fever, and lactatemia.^1,2,4^ There is a dose-dependent nature of the toxicity and most cases occurred in patients who were administered propofol for >48 h at doses exceeding 4 mg/kg/h.^4^

PRIS was first described in the 1990s in children who were sedated with propofol via continuous infusion in the intensive care unit (ICU).^5,6^ Virtually all developed bradyarrhythmias, cardiac conduction abnormalities, and profound myocardial failure.^5,7^ Importantly, cardiac involvement was associated with mortality in most of these pediatric cases.^5–7^ The syndrome was also recently described in a relatively healthy newborn following a single bolus of propofol for induction of anesthesia in which bradycardia and hemodynamic instability were early and prominent features.^8^ Because PRIS can be lethal, the US Food and Drug Administration has warned against propofol use for sedation of pediatric patients in the ICU setting.^9^

The exact pathophysiology of PRIS has yet to be clearly defined. Evidence, however, exists to suggest that propofol interacts with various components of the electron transport chain (ETC).^1,2^ For example, propofol inhibits Complexes I and IV at different concentrations and potentially interferes with the redox activity of coenzyme Q (CoQ).^4,10–12^ In addition, propofol uncouples non-phosphorylating mitochondria by enhancing proton leak.^10^ Furthermore, it has been suggested that PRIS might result from an acquired fatty acid oxidation defect due to carnitine palmitoyltransferase I inhibition.^13^

Despite the fact that children are vulnerable to developing PRIS and that young age may be a risk factor, little information is available regarding the mechanism of propofol-mediated toxicity during development.^6^ In addition, the toxicological effects of propofol in the context of PRIS have never been assessed in immature mitochondria. PRIS is considered to be pediatric in nature when it occurs in children under 16 years of age.^1^ Although this is a wide age range, the majority of pediatric cases have occurred in infants and younger children.^7^ Thus, we aimed to determine the mechanism of propofol toxicity in newborn mice, modeling for exposure during human infancy.^14–16^ We focused on cardiomyocyte mitochondria and the developing heart given the high incidence of cardiac involvement in children who develop PRIS and the association between acquired cardiovascular disturbances and propofol-related mortality.^7^ We hypothesized that propofol would induce discrete defects within immature cardiac mitochondria. Using an in vitro approach, we found that propofol uncoupled immature cardiomyocyte mitochondria by inducing excessive CoQ-sensitive proton leak and interfered with electron transport at CoQ. Importantly, these defects were targetable, providing new insight into the mechanism of propofol toxicity in the developing heart.

## Materials and methods

### Animals

Care was in accordance with NIH and CUMC IACUC guidelines. Six–8-week-old C57Bl/6N breeding pairs were acquired (Charles River, Wilmington, MA) to yield male pups. P10 modeled a timepoint in human infancy.^14–16^

### Mitochondrial isolation

Cardiac ventricles were harvested, homogenized in ice-cold buffer (225 mM mannitol, 75 mM sucrose, 1 mM EGTA, 5 mM HEPES-KOH (pH 7.2) and 1 mg/mL of fatty-acid-free bovine serum albumin (BSA)), and centrifuged (1100 × *g*) for 5 min at 4 °C. The supernatant was centrifuged (18,500 × *g*) for 10 min at 4 °C using 15 vol% Percoll gradient. Pellet was suspended in buffer and centrifuged (10,000 × *g*) for 10 min at 4 °C. Pellet was resuspended and protein concentration was determined.

### In vitro exposure

Mitochondria were exposed to propofol (Diprivan, Fresenius Kabi, Lake Zurich, Illinois) in vitro (50, 100, 200, or 400 µM). Concentrations were chosen based on the therapeutic range (5–30 µM in plasma and 60–90 µM in tissue).^2,17^ Although propofol levels reach 400 µM in the heart following a single bolus, concentrations chosen for in vitro exposure modeled supratherapeutic propofol exposures, generally within the toxic range.^4,18^ Injectable emulsion permitted assessment of the clinical formulation, recognizing it is unknown if emulsion reaches mitochondria without a change in composition following injection. In vitro intralipid (10%, equal volume as 400 µM propofol, Sigma-Aldrich, St. Louis, MO) exposure controlled for propofol solvent. A separate cohort was assessed in the absence of propofol or lipid to serve as non-exposed controls.

### Mitochondrial oxygen consumption

Mitochondria (50 µg) were assessed in 0.5-mL respiration buffer (200 mM sucrose, 25 mM KCl, 2 mM K_2_HPO_4_, 5 mM HEPES-KOH (pH 7.2), 5 mM MgCl_2_, 0.2 mg/mL BSA) with a Clark-type electrode (Oxytherm, Hansatech, UK) using Complex I-dependent (10 mM glutamate, 5 mM malate) or Complex II-dependent (10 mM succinate with 5 µM rotenone) substrates at 32 °C. State 3 was initiated with ADP (200 µM). State 4 was induced with oligomycin (2.5 µg/mL) and uncoupled state 3 respiration was initiated using dinitrophenol (DNP; 70 µM).

### Modular kinetics

Proton leak, substrate oxidation, and ATP turnover were measured in the presence of 400 μM propofol, equal volume 10% intralipid, or in non-exposed mitochondria.^19^ Oxygen consumption and mitochondrial membrane potential (ΔΨ) were measured in mitochondria (0.1 mg) in 1-mL respiration buffer containing 80 ng/mL nigericin (to collapse ΔpH) and 5 μM rotenone at 37 °C. ΔΨ was quantified using an electrode selective for tetraphenylphosphonium (World Precision Instruments, Sarasota, FL).^20^ Respiration was initiated using 5 mM succinate. For proton leak, respiration was induced with oligomycin (2.5 μg/mL) and titrated with up to 2 mM malonate. For substrate oxidation, state 4 was induced with oligomycin (2.5 μg/mL) and titrated with DNP (up to 100 μM). For ATP turnover, state 3 was induced with an ADP-regenerating system (100 μM ATP, 20 mM glucose, and 10 units/mL hexokinase [baker’s yeast, Sigma-Aldrich]) and titrated with up to 200 μM malonate.

In separate experiments, oligomycin (2.5 µg/mL) was added following ADP (200 µM) to test for reverse activity of the ATP synthase. To determine leak source during state 4, cyclosporine A (CsA, 1 μM), carboxyatractyloside (cAT, 1 μM), and guanosine diphosphate (GDP, 0.75 mM) were added to inhibit the mitochondrial permeability transition pore (mPTP), the adenine nucleotide translocase (ANT), and uncoupling proteins (UCPs), respectively.^21,22^

### Steady-state ETC enzyme activity

Citrate synthase activity was determined (Sigma-Aldrich CS0720) in isolated mitochondria using spectrophotometry. Inhibitor-sensitive respiratory chain complex activities were measured in 1-mL volume spectrophotometrically in the presence of 400 μM propofol or equal volume 10% intralipid or in non-exposed mitochondria and normalized to citrate synthase activity.^23,24^ Rotenone-sensitive Complex I-specific activity was measured in mitochondria (40 µg) with 4.8 mM^−1^ cm^−1^ as the extinction coefficient of NADH at 340 nm using a reference wavelength of 380 nm. 2-Thenoyltrifluoroacetone-sensitive Complex II activity was determined in mitochondria (40 µg) using 19.1 mM^−1^ cm^−1^ as the extinction coefficient of 2,6-dichlorophenolindophenol at 600 nm. For Complexes III and IV, inhibitor-sensitive first-order rate constants were calculated in mitochondria (4 and 2 µg, respectively) using 18.5 mM^−1^ cm^−1^ as the extinction coefficient of cytochrome c at 550 nm. Oligomycin-sensitive Complex V specific activity was measured in mitochondria (40 µg) with 6.2 mM^−1^ cm^−1^ as the extinction coefficient of NADH at 340 nm. Rotenone-sensitive Complex I+III linked activity and antimycin A-sensitive Complex II+III linked activity were determined separately in mitochondria (40 µg) with 18.5 mM^−1^ cm^−1^ as the extinction coefficient of cytochrome c at 550 nm.

### Effect of exogenous quinones

Effect of CoQ_0_ (2,3-dimethoxy-5-methyl-*p*-benzoquinone, 100 µM, Sigma-Aldrich) on steady-state Complex II+III activity was determined in propofol-, intralipid-, and non-exposed mitochondria. Menaquinone-4 (100 µM, Sigma-Aldrich) served as control quinone given the lack of electron carrier activity.^25^ Separately, CoQ_0_ and menaquinone-4 (50 µM) were added to respiring mitochondria to determine the effect on oligomycin-induced leak respiration.

### Statistical analysis

Data were assessed for normality by examining histograms and box plots. Statistical analysis was performed using GraphPad Prism 8 (GraphPad Software, La Jolla, CA). Data are presented as means ± SD (unless otherwise indicated). The sample number for each experiment is indicated in the figure legends. Differences between and within exposed cohorts were assessed using a one-way analysis of variance with Tukey’s post hoc test. Two-tailed, unpaired Student’s *t* test compared differences in modular kinetics respiration rates or ΔΨ between groups. Significance set at *P* < 0.05.

## Results

### Toxic concentrations of propofol alter oxygen consumption

**Fig. 1:**
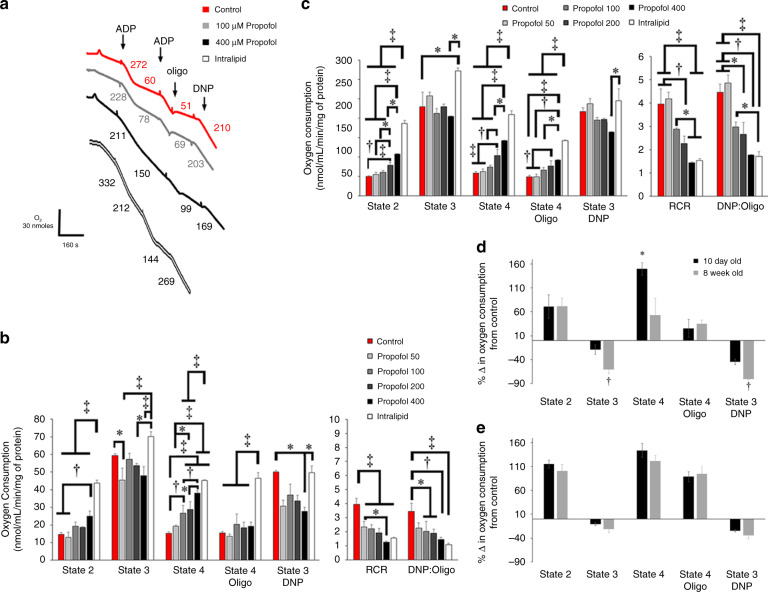
Oxygen consumption in isolated cardiomyocyte mitochondria. **a** Representative tracings for Complex II-dependent oxygen consumption in non-exposed controls or mitochondria exposed to propofol or intralipid are depicted. Numbers following the addition of adenosine diphosphate (ADP), oligomycin (oligo), and dinitrophenol (DNP) indicate oxygen consumption rates (nmol/mL/min/mg mitochondrial protein). Graphical representations of **b** Complex I-dependent oxygen consumption using glutamate/malate and **c** Complex II-dependent oxygen consumption using succinate are shown (*n* = 3–5 per group). Rates of state 2 respiration, state 3 respiration (following the addition of ADP), state 4 respiration, uncoupled state 3 respiration (state 3 DNP), and oligomycin-induced state 4 (state 4 oligo) are indicated. Graphical depiction of respiratory control ratios (RCR) and DNP-to-oligomycin ratios (DNP:oligo) is also shown. Percent change from control values of **d** Complex I-dependent oxygen consumption using glutamate/malate and **e** Complex II-dependent oxygen consumption in 10-day-old and 8-week-old mitochondria exposed to the highest concentration of propofol is depicted. States of respiration are indicated. Values are expressed as means ± SD. *p* Values were calculated by one-way ANOVA for **b**, **c**. Student’s *t* test was calculated for **d**, **e**. *p < 0.05, ^†^*p* < 0.01, ^‡^*p* < 0.001.

To determine in vitro concentration-dependent effects of propofol, we first measured respiration using a Clark-type electrode in mitochondria harvested from newborn mice on postnatal day 10 and young adults at 8 weeks of age. In immature mitochondria, propofol induced dose-dependent increases in state 2 and state 4 respiration for Complex I-dependent substrates and significantly decreased dinitrophenol (DNP)-induced state 3 at the highest concentration (Fig. 1b). For Complex II-dependent substrate, propofol induced dose-dependent increases in state 2, state 4, and oligomycin-induced state 4 respiration (Fig. 1c). There was no effect of propofol on Complex II-dependent state 3 (Fig. 1c). Thus, propofol uncoupled respiration in immature mitochondria by inducing proton leak regardless of substrate and inhibited Complex I-dependent respiration at the highest concentration. This was supported by significant decreases in respiratory control and DNP-to-oligomycin ratios (Fig. 1b, c).

In mature mitochondria, propofol inhibited respiration regardless of substrate, as evidenced by a significant decrease in state 3 or DNP-induced state 3 respiration, and induced leak only with Complex II-dependent substrate (Supplemental Figure). When comparing the change in oxygen consumption from control between immature and mature mitochondria during exposure to the highest concentration of propofol, the directionality of change was equivalent between groups for each state of respiration (Fig. 1d, e). However, the percent change in state 3 and DNP-induced state 3 respiration was significantly greater in 8-week-old cardiomyocyte mitochondria for Complex I-dependent substrates, indicating greater propofol-mediated inhibition (Fig. 1d). With regard to leak, change in state 4 respiration was significantly higher in P10 mitochondria for Complex I-dependent substrates (Fig. 1d). Thus, there were age-dependent differences in mitochondrial respiration during in vitro exposure to toxic concentrations of propofol.

In contrast to propofol, intralipid significantly increased state 2, state 4, and oligomycin-induced state 4 respiration to a greater degree and significantly increased Complex II-dependent state 3 respiration (Fig. 1c). Intralipid also induced an increase in Complex I-dependent state 3 respiration that was significantly higher than propofol rates (Fig. 1b). Thus, intralipid uncoupled newborn mitochondrial respiration by inducing proton leak, however, stimulated respiration to a greater degree than propofol without evidence of inhibition.

### Propofol induces pathological leak and limits substrate oxidation

To define the effects more precisely, we performed modular kinetic analysis.^26^ Modular kinetics is an in-depth quantitative approach that employs the parallel measurement of oxygen consumption with mitochondrial membrane potential (ΔΨ) over a range of potentials to dynamically assess the interconnected processes that relate to the proton motive force.^26^ Thus, the approach permits precise interpretation of changes in proton leak, substrate oxidation, and ATP turnover.^26^

**Fig. 2:**
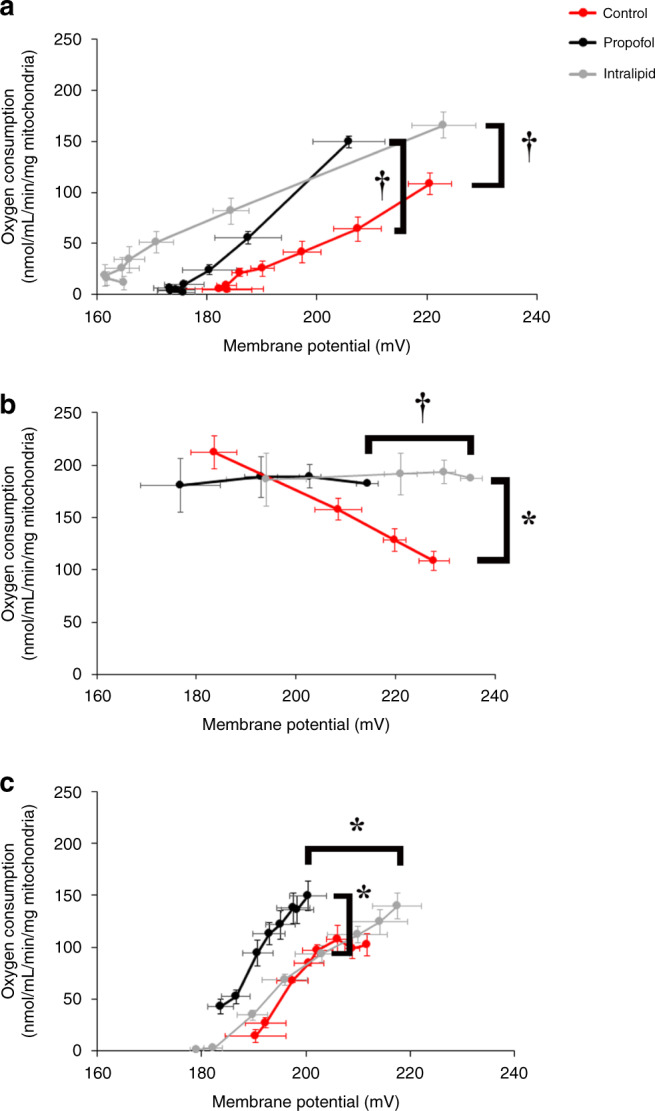
Modular kinetic analysis of isolated cardiomyocyte mitochondria. Oxygen consumption rates using succinate were measured over a range of ΔΨs with and without propofol (400 µM) or intralipid. For proton leak (**a**), state 4 was induced with oligomycin and respiration was titrated with serial additions of malonate. For substrate oxidation (**b**), state 4 was induced with oligomycin and respiration was titrated with serial additions of dinitrophenol. For ATP turnover (**c**), state 3 respiration was induced using an ADP-regenerating system and titrated with serial additions of malonate. *n* = 3–5 per group. Values are expressed as means ± SEM. *p* values for oxygen consumption rate at the highest common ΔΨ were calculated by Student’s *t* test. *p* values were also determined for ΔΨ at the highest common oxygen consumption rate. *p < 0.05, ^†^*p* < 0.01.

With the proton leak module, propofol and intralipid shifted the conductance curves to the left of controls such that the rates of oxygen consumption at the highest common ΔΨ were significantly increased in propofol- and lipid-exposed mitochondria (Fig. 2a). Therefore, propofol and intralipid induced significant proton leak. With substrate oxidation, both propofol and intralipid flattened the curve (Fig. 2b). However, lipid significantly increased the oxygen consumption rate at the highest common ΔΨ relative to controls, while propofol had no significant effect (Fig. 2b). The substrate oxidation curve was left-shifted in propofol-exposed mitochondria with a significantly decreased ΔΨ compared to intralipid (Fig. 2b). Thus, intralipid induced an increase in substrate oxidation that was not observed in propofol-exposed mitochondria. Taken together, the data indicated that both propofol and intralipid increased proton conductance in immature mitochondria. However, intralipid-exposed mitochondria compensated for excessive proton leak by increasing substrate oxidation to generate an adequate ΔΨ, while propofol-exposed mitochondria failed to do so.

**Fig. 3:**
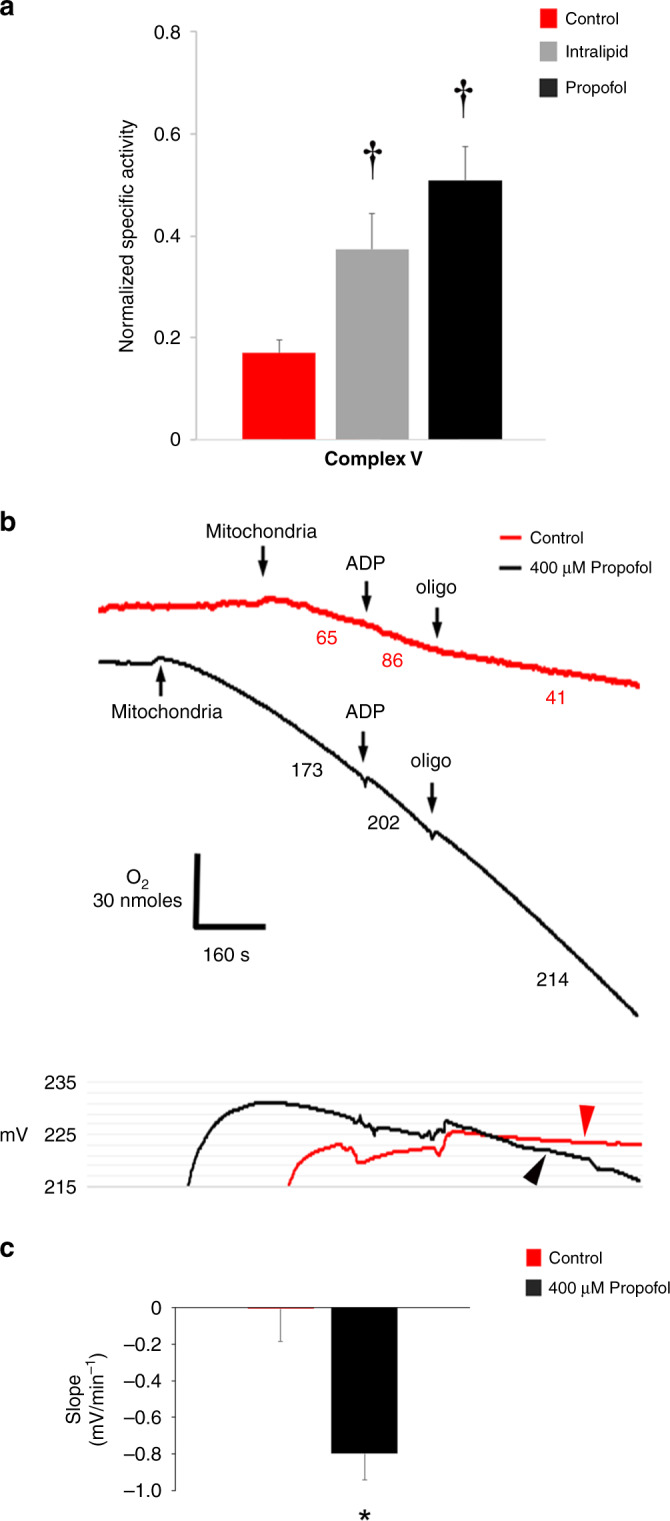
Assessment for the reverse activity of the ATP synthase. **a** Oligomycin-sensitive steady-state hydrolytic activity of Complex V was measured in non-exposed controls and in mitochondria exposed to propofol (400 µM) or intralipid. Values were normalized to citrate synthase activity and expressed as means ± SD (*n* = 4–5 per group). *p* values were calculated by one-way ANOVA. ^†^*p* < 0.01 vs. control. **b** Representative tracings of Complex II-dependent oxygen consumption using succinate are depicted above and tracings of simultaneously measured ΔΨ are shown below (from 3 biological replicates in each group). State 3 was initiated with ADP and state 4 was initiated with oligomycin (oligo). Numbers in the tracings above indicate oxygen consumption rates (nmol/mL/min/mg mitochondrial protein). Non-exposed controls demonstrated increased respiration and fall in ΔΨ following ADP and a decline in respiration along with increased ΔΨ following oligo. ΔΨ remained stable following oligo in controls (red arrowhead). In propofol-exposed mitochondria, ΔΨ persistently declined following oligo (black arrowhead), indicating reverse activity of the ATP synthase. **c** Slope of change in ΔΨ following the addition of oligo in **b** is depicted. Values are means ± SD (*n* = 3 per group). *p* values were calculated by Student’s *t* test. *p < 0.05 vs. control.

With regard to ATP turnover, propofol shifted the curve to the left of control and intralipid-exposed mitochondria and significantly increased mitochondrial respiration at the highest common ΔΨ (Fig. 2c). Thus, propofol increased ATP synthesis, export, and turnover. As with substrate oxidation, ΔΨ was significantly lower in propofol-exposed mitochondria relative to intralipid-exposed mitochondria (Fig. 2c). We hypothesized that the increase in ATP turnover in propofol-exposed mitochondria was due, in part, to reverse activity of the ATP synthase, consuming ATP to pump protons in the setting of compromised ΔΨ.^27^ To test this, we measured the hydrolytic activity of Complex V via spectrophotometry and determined the effect of the ATPase inhibitor, oligomycin, on ΔΨ in during in vitro exposure to propofol. We found significantly increased steady-state hydrolytic activity of Complex V in propofol-exposed mitochondria and ΔΨ steadily declined following the addition of oligomycin (Fig. 3). Thus, propofol-induced ATP synthase reverse activity. However, increased ATP turnover was futile given the inability to defend and maintain the ΔΨ during propofol exposure. The increase in hydrolytic activity of Complex V in intralipid-exposed mitochondria was consistent with the known stimulatory effects of fatty acids on the rate of ATP hydrolysis (Fig. 3a).^28^ Such stimulation is thought to be related to lipid-induced increases in membrane conductance.^28^

### Source(s) of proton leak

**Fig. 4:**
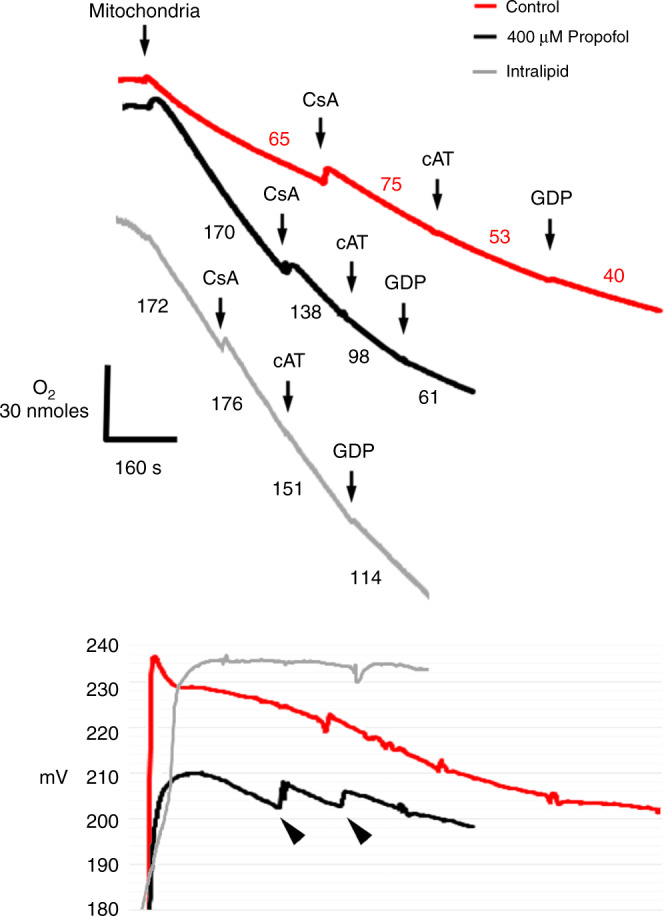
Sources of proton leak. Oligomycin-induced state 4 was initiated using succinate. Representative tracings of oxygen consumption (above) with simultaneous ΔΨ measurement (below) are depicted (from 3 to 4 biological replicates in each group). Numbers indicate oxygen consumption rates (nmol/mL/min/mg mitochondrial protein). Cyclosporine A (CsA), carboxyatractyloside (cAT), and guanosine diphosphate (GDP) were added to specifically inhibit leak via the mitochondrial permeability transition pore (mPTP), the adenine nucleotide translocase (ANT), and uncoupling proteins (UCPs), respectively. Inhibition of leak was identified by a concomitant decrease in oxygen consumption with a rise or stabilization in ΔΨ. Arrowheads indicate persistent leak despite the obvious closure of a leak channel.

Proton leak is largely mediated by the adenine nucleotide translocase (ANT), uncoupling proteins (UCPs), and the mitochondrial permeability transition pore (mPTP).^21,22^ Therefore, we utilized specific inhibitors to delineate the known source(s) of leak during state 4 respiration. Both propofol and intralipid increased proton leak, evidenced by elevated oligomycin-induced state 4 respiration rates (Fig. 4). ΔΨ was lower in the presence of propofol, suggesting a defect in generating an adequate ΔΨ (Fig. 4). In controls, the ANT inhibitor, carboxyatractyloside (cAT), and the UCP inhibitor, guanosine diphosphate (GDP), decreased respiration and stabilized ΔΨ (Fig. 4). Thus, the ANT and UCPs were the predominant sources of physiological leak in controls. In intralipid-exposed mitochondria, of the inhibitors utilized, the greatest effect was seen with GDP (Fig. 4). This indicated that intralipid-induced proton leak was mediated largely via UCPs. It should be noted that persistent leak was evident following the addition of GDP, indicating the existence of other unknown and untested leak sources. CsA, cAT, and GDP each caused a decline in respiration in propofol-exposed mitochondria, suggesting the mPTP, ANT, and UCPs as sources of leak (Fig. 4). However, the persistent fall in ΔΨ without stabilization, especially following the noticeable upward deflection in ΔΨ (indicating closure of leak channel), suggested an unidentified source of propofol-induced pathological leak (Fig. 4).

### Propofol interferes with electron transport at the level of CoQ

**Fig. 5:**
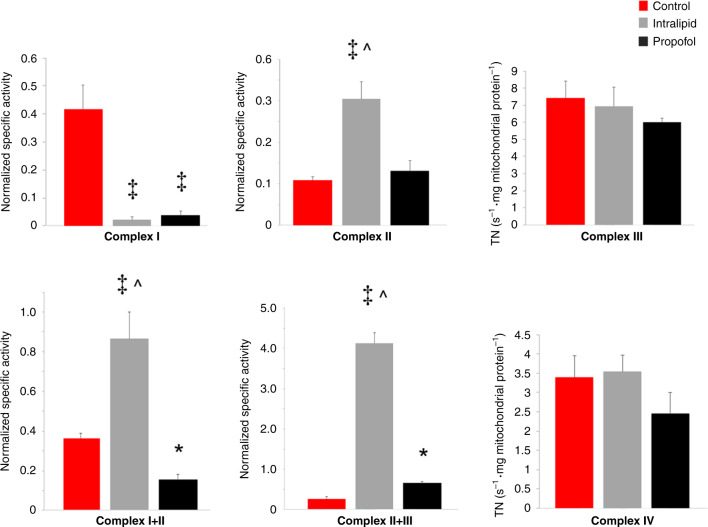
Cardiomyocyte electron transport chain enzyme complex kinetic activity. Steady-state kinetic activities were determined in non-exposed controls and in mitochondria exposed to propofol (400 µM) or intralipid. The specific activities normalized to citrate synthase activity are depicted (*n* = 4–5 per group). Coenzyme Q-dependent respiration was assessed by measuring linked Complex I+III and Complex II+III kinetic activities. First-order rate constants were determined for Complexes III and IV and expressed as turnover number (TN). Values are expressed as means ± SD. *p* values were calculated by one-way ANOVA. *p < 0.05 vs. control, ^‡^*p* < 0.001 vs. control, ^*p* < 0.001 vs. propofol-exposed group.

We next determined the in vitro effects of exposure on the steady-state kinetic activity of the ETC complexes to identify where propofol interfered with substrate oxidation. Propofol and intralipid profoundly inhibited Complex I (Fig. 5). Yet, intralipid significantly increased Complex II activity and markedly enhanced Complex I+III and Complex II+III linked activities (Fig. 5). The combined effect of intralipid on Complex I+III and II+III linked activities indicated stimulation of electron flux at CoQ (especially in the context of Complex I inhibition). Although the effects of propofol on Complex I+III and II+III diverged relative to controls, the linked activities were significantly lower than intralipid values (Fig. 5). Thus, propofol interfered with electron transport at CoQ.

### A CoQ analog increases electron transport and blocks proton leak in propofol-exposed mitochondria

**Fig. 6:**
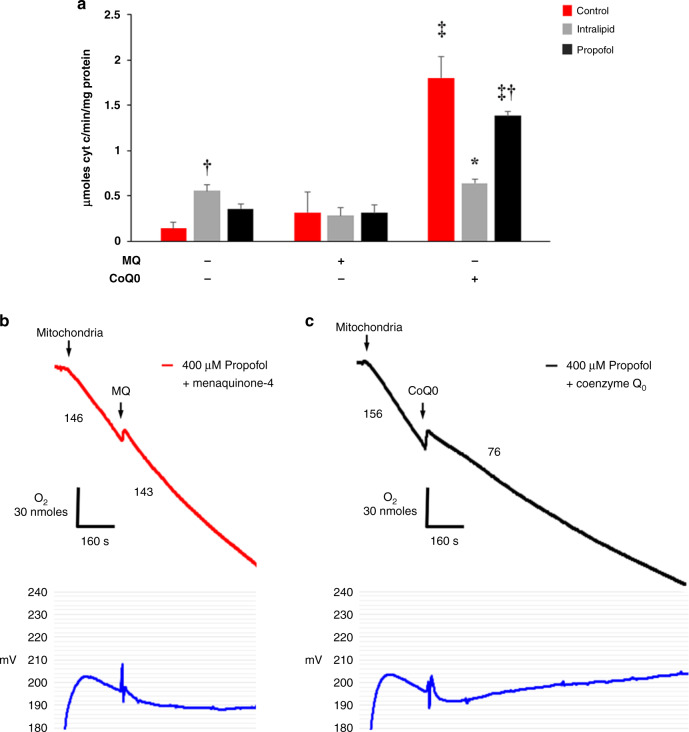
Effect of coenzyme Q_0_ on electron transfer and proton leak. a Linked Complex II+III kinetic activity was measured in non-exposed controls and in mitochondria exposed to propofol (400 µM) or intralipid. Effect of CoQ_0_ was compared with the effects of menaquinone-4 (MQ) and no added quinone (*n* = 4–7 per group). Values are expressed as means ± SD. *p* values were calculated by one-way ANOVA. *p < 0.05 vs. intralipid-exposed MQ-added cohort, control, and propofol-exposed CoQ_0_-added cohorts, ^†^*p* < 0.01 vs. control within quinone treatment group, ^‡^*p* < 0.001 vs. no added quinone, MQ within exposure cohort. **b**, **c** Oligomycin-induced state 4 respiration was initiated using succinate in the presence of propofol (400 µM). Representative tracings of oxygen consumption (above) with simultaneous ΔΨ measurement (below) are depicted (from 3 biological replicates in each group). Numbers indicate oxygen consumption rates (nmol/mL/min/mg mitochondrial protein). **b** MQ or **c** CoQ_0_ were added to assess for the effect on leak.

We next assessed the effect of the ubiquinone analog, CoQ_0_ (2,3-Dimethoxy-5-methyl-*p*-benzoquinone), on steady-state Complex II+III activity during in vitro exposure. We utilized this specific analog because it lacks a side chain, permitting rapid diffusion across the mitochondrial outer membrane.^26^ Menaquinone-4 (MQ) served as a control quinone given that it lacks electron carrier activity.^25^ MQ had no significant effect on Complex II+III activity in any group (Fig. 6a). In contrast, CoQ_0_ significantly increased Complex II+III activity within each exposure cohort in all groups compared to the effect of MQ, lack of added quinone, or both, indicating a CoQ-mediated increase in electron transport (Fig. 6a). Of note, CoQ_0_-treated propofol-exposed mitochondria failed to reach the maximum velocity observed in CoQ_0_-treated controls, suggesting persistent propofol-mediated interference with electron transfer at CoQ (Fig. 6a). Also, Complex II+III activity was significantly lower in CoQ_0_-treated lipid-exposed mitochondria compared with CoQ_0_-treated control and propofol-exposed cohorts (Fig. 6a). This finding, in particular, highlights certain limitations of utilizing the lipid solvent as a control group. Despite these nuances, the findings strengthen the notion that propofol interrupts CoQ-dependent electron transfer and indicate that excess exogenous CoQ can partially abrogate this effect.

Because ubiquinone is a known leak modulator, we assessed the in vitro effect of CoQ_0_ on propofol-induced leak. Propofol induced excessive proton leak as evidenced by high state 4 respiration rates and relatively low ΔΨs (Fig. 6b, c). MQ had minimal effect on the rate of oxygen consumption and the decline in ΔΨ (Fig. 6b). ΔΨ, however, leveled off and stabilized several minutes after the addition of MQ (Fig. 6b). Although this appeared to differ from results depicted in Fig. 3b, ΔΨ stabilization occurred at a relatively low level after a longer period of observation. This likely reflected the effects of excessive proton leak and limited substrate oxidation (not complete inhibition). Thus, we anticipated the plateau of ΔΨ without a complete collapse. In contrast, CoQ_0_ immediately decreased oligomycin-induced state 4 respiration and induced a gradual and steady rise in ΔΨ (Fig. 6c). Thus, CoQ_0_ inhibited proton leak during in vitro propofol exposure. Taken together, the data suggest that propofol causes a pathological leak, in part, via a CoQ-sensitive leak channel and that CoQ_0_ can both increase electron flux and block excessive proton leak in propofol-exposed mitochondria.

## Discussion

Although the exact pathophysiology of PRIS remains unknown, there is general consensus that an acquired defect in mitochondrial function underlies this rare but potentially lethal process. Mounting evidence suggests that propofol inhibits and interferes with multiple enzymes and components within the ETC and disrupts fatty acid oxidation.^1,2,4,10–13^ However, the studies that provided such mechanistic insight focused largely on adult animals, evaluating only mature mitochondria from fully developed tissues and organs. Therefore, in this work, we assessed the toxicological effects of propofol in immature mitochondria given that children are vulnerable to developing PRIS and young age is a risk factor. We specifically studied cardiomyocyte mitochondria because of the high incidence of cardiac involvement in children who develop PRIS and the association between acquired cardiovascular disturbances and propofol-related mortality.

We found that toxic concentrations of propofol uncoupled immature cardiomyocyte mitochondria by inducing a dose-dependent increase in proton leak. We identified the ANT, UCPs, and the mPTP as sources of leak during propofol exposure and elucidated the important role of a CoQ-sensitive leak channel. Further in-depth analysis revealed that propofol also interfered with electron transport at the level of CoQ, resulting in a relative defect in substrate oxidation. The combination of excessive proton leak and limited capacity to oxidize substrate prevented propofol-exposed cardiomyocyte mitochondria from generating an adequate ΔΨ. This impairment was profound enough to render reverse ATP synthase activity futile in an attempt to maintain and defend the ΔΨ.

Our results are consistent with findings from a previous investigation of mature mitochondria that found propofol to uncouple non-phosphorylating rat liver mitochondria by enhancing proton leak and other work that demonstrated propofol-mediated impedance of electron flow through the ETC, largely at the level of CoQ.^4,10^ However, no prior study evaluated the effect of propofol on both mature and immature mitochondria. Although we focused mostly on developing mitochondria in this work, we found propofol to affect actively respiring in newborn and young adult mitochondria differently. Further investigation will certainly be necessary to determine whether there are, indeed, age-dependent differences in response to propofol. Despite the unknowns, however, our findings provide some insight into why the young may be vulnerable to developing PRIS.

It is well known that the ANT mediates basal proton conductance and UCP-induced leak can be activated by fatty acids.^29^ However, finding the mPTP to be a source of leak only in propofol-exposed mitochondria could carry an importance. The mPTP is known to play an important physiological role in the developing heart.^30^ Closure of the mPTP induces a switch from anaerobic glycolysis to oxidative phosphorylation in developing cardiomyocytes, establishing the ΔΨ and triggering cellular differentiation.^30–32^ Cardiac mitochondria undergo maturation within the first few weeks of life, yet, the probability of mPTP opening remains high in the postnatal period, rendering the neonatal heart vulnerable to injury.^30^ Although the exact factors involved in regulating the mPTP during development are unknown, molecules, such as ubiquinone, serve as modulators.^33^ Biosynthesis of CoQ is low in infancy and increases in young adulthood.^34^ Thus, low CoQ levels in the context of a propofol-CoQ interaction could explain why the mPTP opens to enhance proton leak in the developing heart. This notion will need to be tested in future work.

In addition to the mPTP, we identified a CoQ-sensitive leak channel as an important source of pathological proton leak in propofol-exposed mitochondria. Although we have yet to determine the exact proteinaceous identity of this specific channel, the CoQ-sensitive leak channel caused excessive proton conductance and compromised ΔΨ during propofol exposure. Evidence for this important role was found in the ability of the quinone analog, CoQ_0_, to block propofol-induced leak on its own and cause a gradual and steady rise in ΔΨ. Such an increase in ΔΨ only occurred with CoQ_0_ and was not observed with any other leak channel inhibitor. This indicated that CoQ_0_ inhibited the pathological proton leak, abrogated the defect in substrate oxidation, or both.

Newborn cardiomyocytes utilize lactate and glucose as metabolic substrates but undergo a switch to fatty acids in the postnatal period.^35,36^ Here we found that intralipid stimulated mitochondrial respiration, increased substrate oxidation, and enhanced electron flux at the level of CoQ. This increase in substrate utilization permitted intralipid-exposed mitochondria to compensate for the fatty acid-mediated increase in proton leak in order to generate an adequate ΔΨ. Such biological activity, however, could confound interpretation of the effects of propofol and limits the utility of intralipid as a control vehicle. Intralipid contains soybean oil (a refined mixture of long-chain fatty acids, such as linoleic acid and oleic acid) and glycerol. Thus, it is likely that intralipid activated the fatty acid β-oxidation pathway to reduce CoQ directly via electron transfer flavoprotein:ubiquinone oxidoreductase or enhance electron flux through CoQ via glycerol-3-phosphate dehydrogenase-dependent respiration.^37,38^ Although the propofol emulsion contains the same formulation of soybean oil and glycerol as intralipid, exposure to propofol caused a relative impairment in substrate oxidation and modulated electron flow through CoQ. In the setting of excessive proton leak, this limitation in the ability to oxidize substrate prevented propofol-exposed mitochondria from generating an adequate ΔΨ. The propofol-induced defect likely involved modulation of one or more of the enzymes within the fatty oxidation pathway, the glycerol-3-phosphate dehydrogenase pathway, or both. Alternatively, the effect on electron flux through CoQ could have involved a direct interaction between propofol and ubiquinone itself. Importantly, exogenous CoQ_0_ enhanced quinone-dependent electron transfer during propofol exposure to partially abrogate the defect.

The dual effects of CoQ_0_ on proton leak and electron flux in propofol-exposed mitochondria are interesting and intriguing. In addition to providing experimental insight, the biological effects establish a foundation for us to develop quinone analogs as novel therapeutics to treat or prevent propofol toxicity in the developing heart. Importantly, our findings with CoQ_0_ support prior work demonstrating that CoQ_10_ prevented propofol-induced cytotoxicity in human induced pluripotent stem cell-derived cardiomyocytes and partially protected T67 and HeLA cells.^39,40^ Thus, future work will focus on targeting propofol-mediated toxicity in the developing heart with CoQ_0_ and related compounds. We plan to extend our investigation using isolated perfused heart preparation and in vivo modeling. Ultimately, we hope to be able to prevent the life-threatening toxicity of propofol in infants and children.

## Supplementary information

Supplementary figure
