# Supplementary material for: Propofol toxicity in the developing mouse heart mitochondria
Source: Pediatr Res. 2022 Feb 16;92(5):1341–9. doi: 10.1038/s41390-022-01985-1 (PMC9378757; doi:10.1038/s41390-022-01985-1)

Supplemental Figure. Oxygen Consumption in 8 week old Cardiomyocyte Mitochondria. (A) Representative tracings for Complex II-dependent oxygen consumption in mitochondria with or without propofol. Numbers following addition of adenosine diphosphate (ADP), oligomycin (oligo), and dinitrophenol (DNP) indicate rates of oxygen consumption (nmol•mL^{-1}•min^{-1}•mg mitochondrial protein^{-1}). Graphical representations of (B) Complex I-dependent oxygen consumption using glutamate/malate and (C) Complex II-dependent oxygen consumption using succinate are shown (n = 3-4 per exposure cohort). Rates of state 2 respiration (following addition of substrate), state 3 respiration (following addition of ADP), state 4 respiration, uncoupled state 3 respiration (state 3 DNP), and oligomycin-induced state 4 (state 4 oligo) are indicated. Graphical depiction of respiratory control ratios (RCR) and DNP-to-oligomycin ratios (DNP:oligo) are also shown. Values are expressed as means ± SD. p values were calculated by one-way ANOVA. *$p < 0.05$, †$p < 0.01$, ‡ $p < 0.001$.

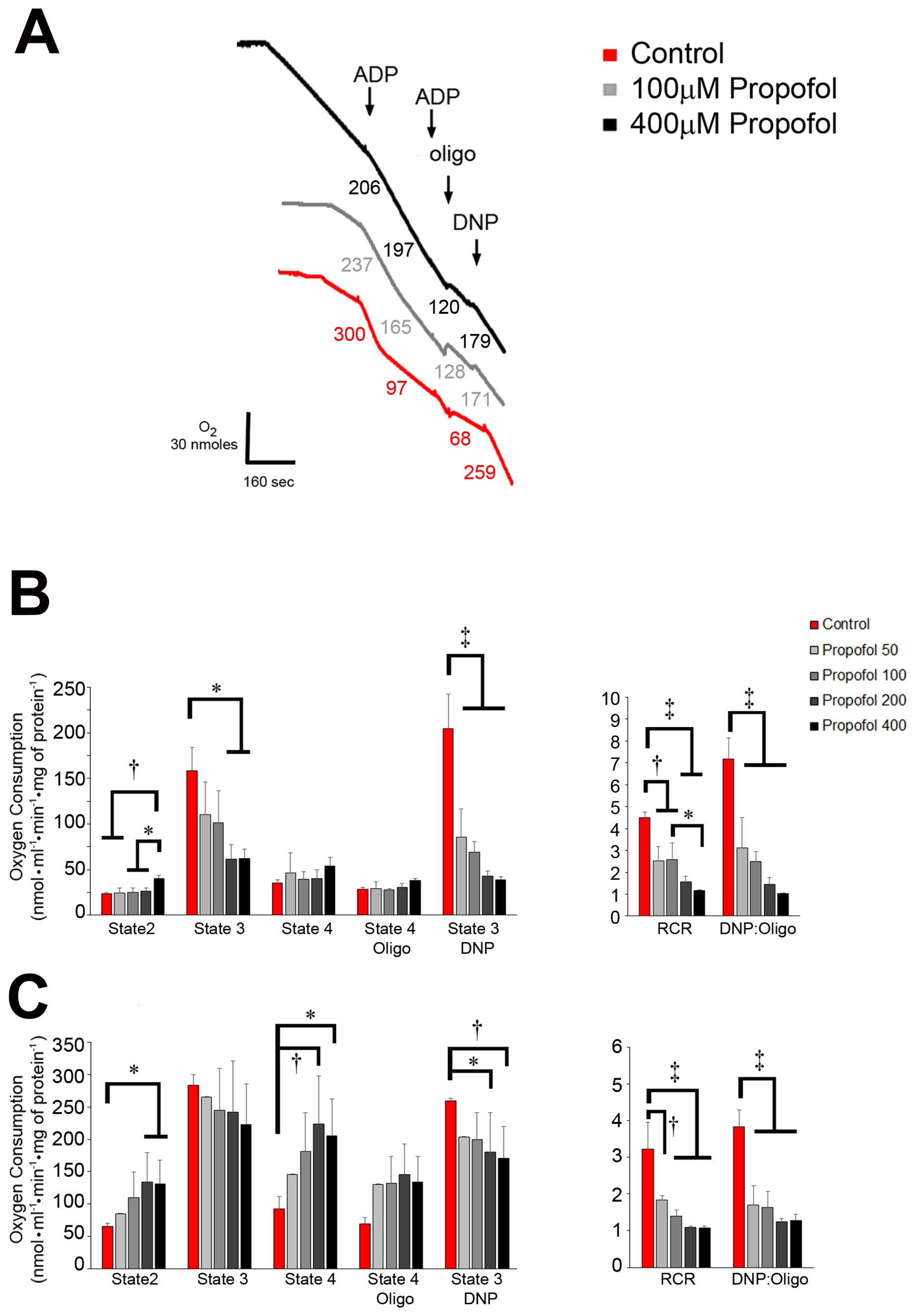

Supplement: Supplementary file 1 — Supplementary figure [file 41390_2022_1985_MOESM1_ESM.pdf]
